# Effect of IL-15 addition on asbestos-induced suppression of human cytotoxic T lymphocyte induction

**DOI:** 10.1186/s12199-021-00967-9

**Published:** 2021-04-19

**Authors:** Naoko Kumagai-Takei, Yasumitsu Nishimura, Hidenori Matsuzaki, Suni Lee, Kei Yoshitome, Tatsuo Ito, Takemi Otsuki

**Affiliations:** 1 Department of Hygiene, Kawasaki Medical School, Kurashiki, 701-0192 Japan; 2 Department of Life Sciences, Faculty of Life and Environmental Sciences, Prefectural University of Hiroshima, 727-0023, Shobara, Japan

**Keywords:** Asbestos, CTL, IL-15, Proliferation, Granzyme B

## Abstract

**Background:**

Asbestos fibers possess tumorigenicity and are thought to cause mesothelioma. We have previously reported that exposure to asbestos fibers causes a reduction in antitumor immunity. Asbestos exposure in the mixed lymphocyte reaction (MLR) showed suppressed induction of cytotoxic T lymphocytes (CTLs), accompanied by a decrease in proliferation of CD8^+^ T cells. Recently, we reported that asbestos-induced suppression of CTL induction is not due to insufficient levels of interleukin-2 (IL-2). In this study, we continue to investigate the mechanism responsible for the effect of asbestos fibers on the differentiation of CTLs and focus on interleukin-15 (IL-15) which is known to be a regulator of T lymphocyte proliferation.

**Methods:**

For MLR, human peripheral blood mononuclear cells (PBMCs) were cultured with irradiated allogenic PBMCs upon exposure to chrysotile B asbestos at 5 μg/ml for 7 days. After 2 days of culture, IL-15 was added at 1 ng/ml. After 7 days of MLR, PBMCs were collected and analyzed for phenotypic and functional markers of CD8^+^ T cells with fluorescence-labeled anti-CD3, anti-CD8, anti-CD45RA, anti-CD45RO, anti-CD25, and anti-granzyme B antibodies using flow cytometry. To examine the effect of IL-15 on the expression level of intracellular granzyme B in proliferating and non-proliferating CD8^+^ lymphocytes, PBMCs were stained using carboxyfluorescein diacetate succinimidyl ester (CFSE) and then washed and used for the MLR.

**Results:**

IL-15 addition partially reversed the decrease in CD3^+^CD8^+^ cell numbers and facilitated complete recovery of granzyme B^+^ cell percentages. IL-15 completely reversed the asbestos-induced decrease in percentage of granzyme B^+^ cells in both non-proliferating CFSE-positive and proliferating CFSE-negative CD8^+^ cells. The asbestos-induced decrease in the percentage of CD25^+^ and CD45RO^+^ cells in CD8^+^ lymphocytes was not reversed by IL-15.

**Conclusion:**

These findings indicate that CTLs induced upon exposure to asbestos possess dysfunctional machinery that can be partly compensated by IL-15 supplementation, and that IL-15 is more effective in the recovery of proliferation and granzyme B levels from asbestos-induced suppression of CTL induction compared with IL-2.

## Background

Exposure to asbestos can lead to tumor diseases such as malignant mesothelioma and lung cancer [1, 2]. Although the use of asbestos has now been prohibited in 55 countries, these prohibitions have only recently been enacted and therefore have not yet had a measureable impact on the incidence of asbestos-related diseases. Asbestos exposure in the developed world has been greatly reduced or eliminated in industrial operations; however, exposure still occurs from asbestos remaining in existing buildings, old industrial sites and from naturally occurring asbestos [3]. There are numerous countries in Asia that continue to mine, import, and use this fiber. Although the first phase to ban the use of asbestos in Japan was announced in 2004, asbestos-related disease is widespread [4]. Japan is currently experiencing an increase in mesothelioma, and rates are predicted to peak sometime between 2030 and 2039 [3]. In the USA, despite the decrease in age-adjusted mesothelioma incidence rates in the past decades, approximately 3000 incident cases of mesothelioma are registered each year. The overall number of new cases and of deaths per year caused by mesothelioma continues to steadily increase in many countries where populations are becoming older since mesothelioma affects mostly older people. The highest age-standardized incidence rates in 2018 were observed in the USA, Australia, Russia, Western Europe, Turkey, South Africa, and Argentina according to the World Health Organization [5].

Mutagenicity or carcinogenicity of asbestos is believed to cause malignant mesothelioma. Chromosomal changes, DNA damage, and oxidative DNA lesions occur in mesothelial cells which cultured upon exposure to asbestos fibers at cytotoxic concentrations [6, 7]. The onset of malignant mesothelioma is not rapid following exposure of asbestos, but rather requires a long elapsed time [8–10]. This suggests the possibility that the body might be protected from malignant mesothelioma by antitumor immunity, functional insufficiency of which might be caused by exposure to asbestos and related with the onset of mesothelioma. In our previous study based on this hypothesis, it was found that asbestos exposure changes the expression pattern of activating receptors on human NK cells and the functions of CD4^+^ T cells [11, 12].

Previously, we reported that asbestos exposure suppressed the differentiation of human mature cytotoxic T lymphocytes (CTLs) in the mixed lymphocyte reaction (MLR) and was accompanied by a decrease in the proliferation of immature CTLs [13]. CD8^+^ lymphocytes in culture following exposure to asbestos showed impaired cytotoxicity with decreases in the proliferation and percentage of CD25^+^ and CD45RO^+^ cells in CD8^+^ lymphocytes and an increase in percentage of CD45RA^+^ cells compared with control cultures. Recently, we focused on investigating the mechanism of the asbestos-induced suppressed differentiation of mature CTLs with the decrease in the proliferation of CD8^+^ lymphocytes and demonstrated that the phenomenon was not attributed to insufficient production of interleukin-2 (IL-2) since exogenous supplementation of IL-2 could augment the cytotoxicity of asbestos-exposed CD8^+^ lymphocytes and percentage of granzyme B^+^ cells but did not restore any other parameters [14]. IL-2 and IL-15 share some functions as a consequence of their utilization of the IL-2Rβ subunit and common γ-chain [15]. It is also considered that interleukin-15 (IL-15) is involved in initiation of the T cell response to an antigen presented by dendritic cells (DCs), such that substantial T cell clonal expansion is driven following T-cell receptor ligation of peptide–MHC, after which time IL-15 plays a role in the generation and maintenance of memory T cells [16]. Therefore, in the present study, we focused on investigating the effect of IL-15 addition on asbestos-induced suppression of mature CTL differentiation with decreased proliferation of immature CTLs. We set out to determine whether IL-15 addition might reverse the suppressed induction of CTLs following exposure to asbestos. The production of IL-15 in the MLR was examined, and the effect of adding IL-15 to the culture at 1 ng/ml on the second day of the MLR was investigated with respect to suppressed CTL differentiation in cultures exposed to chrysotile B (CB) asbestos.

## Methods

### Cell preparation

PBMCs were separated from heparinized blood obtained from healthy donors using a Ficoll-Hypaque density gradient (Separate-L, Muto Pure Chemicals Co. Ltd., Tokyo, Japan). Freshly isolated PBMCs were suspended in RPMI 1640 medium (Sigma-Aldrich, St. Louis, MO, USA) supplemented with 10% heat-inactivated fetal bovine serum (Medical and Biological Laboratories Co., Ltd., Nagoya, Japan), 100 μg/ml streptomycin, and 100 U/ml penicillin (Meiji Seika Pharma Co., Ltd., Tokyo, Japan), as previously described [14].

### MLRs

For the MLRs, PBMCs were stimulated with allogenic PBMCs (PBMCs:allogenic PBMCs ratio = 1.5 × 10^5^:5.0 × 10^4^), that had been treated with irradiation of 40 Gy according to a previous method [17]. Some of the MLRs were performed in the presence of CB asbestos at 5 μg/ml. Following 2 days of the MLRs with CB, IL-15 (Peprotec, Rocky Hill, NJ, USA) was added at 1 ng/ml for 5 days. International Union Against Cancer (UICC) standard CB was kindly provided by the Department of Occupational Health at the National Institute for Occupational Health of South Africa [18].

### ELISA

Culture supernatants were collected at days 0, 2, 4, and 7 after the MLR, and assayed for the production of IL-15 using a Quantikine ELISA kit (R&D Systems, Inc. Minneapolis, MN, USA).

### Assay for cell surface expression levels

To examine the expression level of molecules on the cell surface, cells harvested after the MLR were washed with PBS containing 2% FBS and then stained with the following antibodies (Abs): phycoerythrin-cychrome 5 (PC5)-conjugated anti-CD8 (Beckman Coulter, Inc., Brea, CA, USA) and fluorescein isothiocyanate (FITC)-conjugated anti-CD3, phycoerythrin (PE)-conjugated anti-CD4, FITC-conjugated anti-CD25 (Becton Dickinson, Franklin Lakes, NJ, USA), PE-conjugated anti-CD45RA, or PE-conjugated anti-CD45RO (BioLegend, San Diego, CA, USA) at room temperature in the dark for 30 min. Cells were then washed with PBS containing 2% FBS and resuspended in 0.3 ml of PBS containing 2% FBS for analysis by flow cytometry (FCM) using FACS Calibur^TM^ (Becton Dickinson) as previously described [14].

### Assay for intracellular expression levels

To examine the expression level of intracellular granzyme B, cells were harvested after the MLR, and surfaces were stained with PC5-conjugated anti-CD8 Ab as described above. Surface-stained cells were washed with PBS containing 2% FBS and then fixed with 3.7% formaldehyde for 15 min. Fixed cells were washed with PBS containing 2% FBS. Fixed cells were permeabilized with 0.1% Triton 100 and stained with R-phycoerythrin (RPE)-conjugated anti-granzyme B Ab (AbD Serotec, Oxford, UK) at room temperature in the dark for 30 min as previously described [14]. Cells were then washed and resuspended as described above. The percentage of cells positive for each parameter was analyzed using FCM.

### Assay for granzyme B production in proliferating and non-proliferating CD8^+^ lymphocytes

To examine the effect of IL-15 on the expression level of intracellular granzyme B in proliferating and non-proliferating CD8^+^ lymphocytes, PBMCs were stained using CFSE (Molecular Probes, Inc., Eugene, OR, USA) and then washed and used for the MLR as previously described [14]. Following the MLR, cells were harvested and stained with PC5-conjugated anti-CD8 and RPE-conjugated anti-granzyme B Abs as described above. The percentage of granzyme B^+^ cells in proliferating CFSE-negative CD8^+^ lymphocytes or non-proliferating CFSE-positive CD8^+^ cells was analyzed using FACSAria^TM^ (Becton Dickinson).

### Statistical analysis

Significance of difference (*p* < 0.05) was determined using an analysis of variance with the post hoc test of Student-Newman-Keuls or paired Student’s *t* test.

## Results

### Effect of chrysotile B asbestos exposure on secreted production of IL-15 during the MLR

**Fig. 1:**
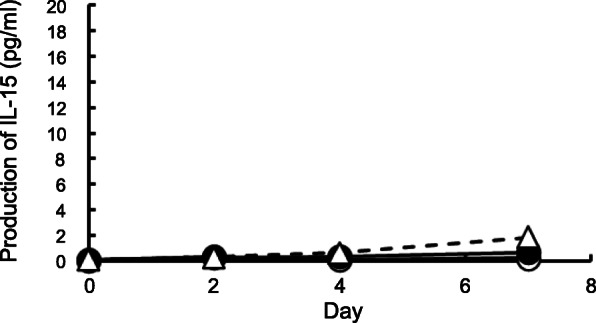
Production of IL-15 during the MLR following exposure to asbestos. Levels of IL-15 in the supernatant of cultures was determined by ELISA at 0, 2, 4, and 7 days after the MLR. Culture supernatants were harvested from the three groups, representing no stimulation (open circle), allogenic stimulation (closed circle), and CB exposed with allogenic stimulation (open triangle). Data represent the mean from four independent experiments using PBMCs from three individuals

Supernatants from cultures of peripheral blood mononuclear cells (PBMCs) with allogenic stimulation in the absence or presence of CB asbestos were harvested and compared for their capacity to produce IL-15 at days 2, 4, and 7 after the MLR. For part of the cultures, PBMCs were cultured alone and supernatants were used as a group without allogenic stimulation. However, secreted IL-15 could not be detected even in the control culture with allogenic stimulation as well as the group without allogenic stimulation at days 2, 4, and 7 in all of the 4 experiments (Fig. 1). In the CB-exposed cultures, production of IL-15 was also not detected. Thus, there were no measurable IL-15 levels in any of the culture supernatants regardless of the absence or presence of exposure to CB. These results indicate the possibility that IL-15 was not produced in these cultures at the level detectable by ELISA.

### Effect of IL-15 on percentage and number of CD3^+^CD8^+^, CD3^+^CD4^+^, and CD3-negative cells in PBMCs

**Fig. 2:**
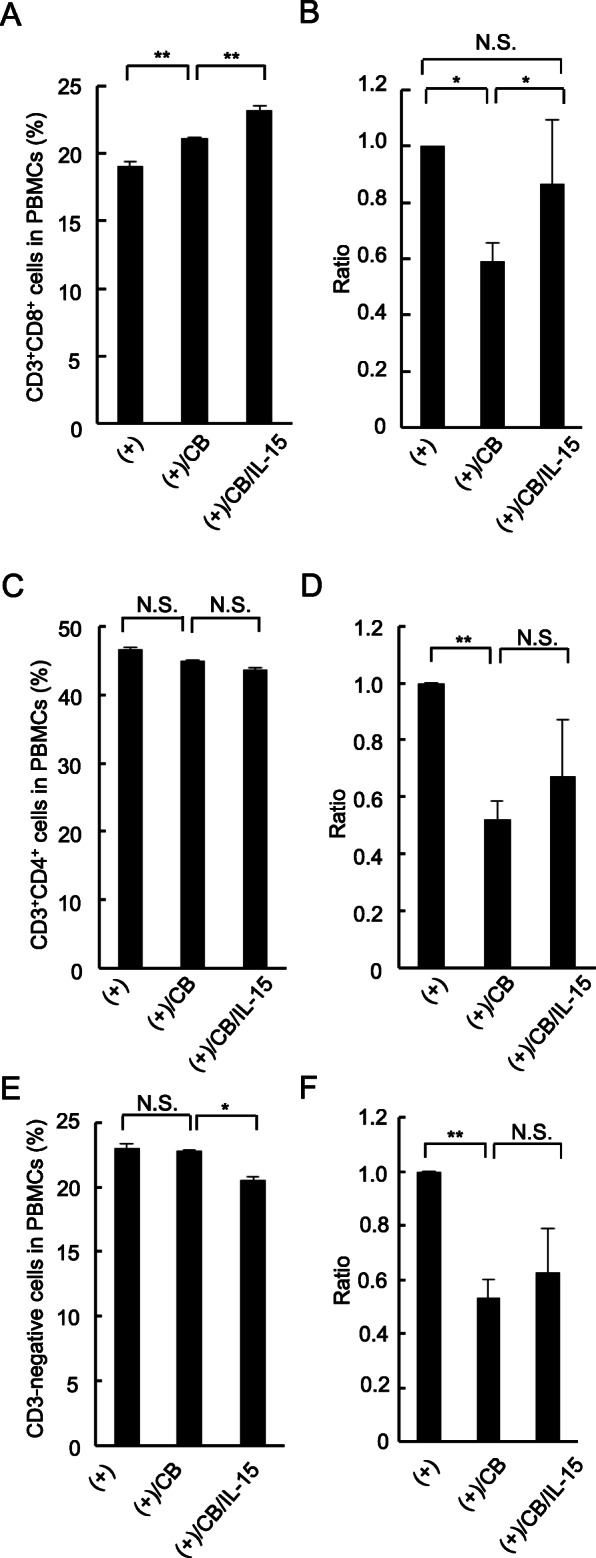
The percentage and number of CD3^+^CD8^+^, CD3^+^CD4^+^, and CD3-negative cells during the induction of effector CTL by MLR. PBMCs were cultured with stimulation of irradiated allogenic PBMCs under exposure to asbestos with or without addition of IL-15. The percentage of CD3^+^CD8^+^, CD3^+^CD4^+^, and CD3-negative cells in harvested PBMCs was measured by FCM (**a**, **c**, and **e**), and the cell number of those per 10 well was calculated and shown as the ratio to the value of control group which stimulated allogenically but not exposed to asbestos or supplemented with IL-15 (**b**, **d**, and **f**). Data are obtained from three independent experiments and are shown as the mean + SD. Significant differences are marked with asterisks (^*^p < 0.05, ^**^p < 0.01). No significant difference is indicated by N.S.. The control group is represented by (+), the group cultured with allogenic PBMCs under exposure to CB is represented by (+)/CB, and the group cultured with allogenic PBMCs under CB exposure with IL-15 is represented by (+)/CB/IL-15

IL-15 was added into the asbestos-exposed culture at day 2 after the MLR, since our previous study showed that the number of carboxyfluorescein diacetate succinimidyl ester (CFSE)-negative cells, that is cells which are proliferating or reaching the end of proliferation, increased markedly in CD8^+^ lymphocytes stimulated with allogenic PBMCs from day 6 to day 7 of the MLR [13]. At day 7, cells were harvested and the percentage and number of CD3^+^CD8^+^ cells were measured. While the percentage of CD3^+^CD8^+^ cells did not decrease (Fig. 2a), exposure to CB caused a significant decrease in the number of CD3^+^CD8^+^ cells, as we previously reported (Fig. 2b). The addition of IL-15 significantly restored the asbestos-induced decrease in the number of CD3^+^CD8^+^ cells (Fig. 2b), which differed from the effect of IL-2 addition [14]. However, the mean value of the IL-15-treated group was intermediate between the groups with and without CB exposure, meaning that IL-15 addition did not completely restore cell numbers. Although the percentage of CD3^+^CD4^+^ and CD3-negative cells as well as CD3^+^CD8^+^ cells did not decrease (Fig. 2c and e), exposure to CB caused a significant decrease in the number of CD3^+^CD4^+^ and CD3-negative cells (Fig. 2d and f). The addition of IL-15 significantly restored the asbestos-induced decrease in the number of CD3^+^CD8^+^ cells, but not CD3^+^CD4^+^ or CD3-negative cell numbers (Fig. 2d and f). These results indicate that exogenously added IL-15 could, to some extent, complement the effect on CB-exposed cultures to maintain the number of CD3^+^CD8^+^ cells.

### Effect of IL-15 on differentiation of naïve CD8^+^ T cells into CTLs

**Fig. 3:**
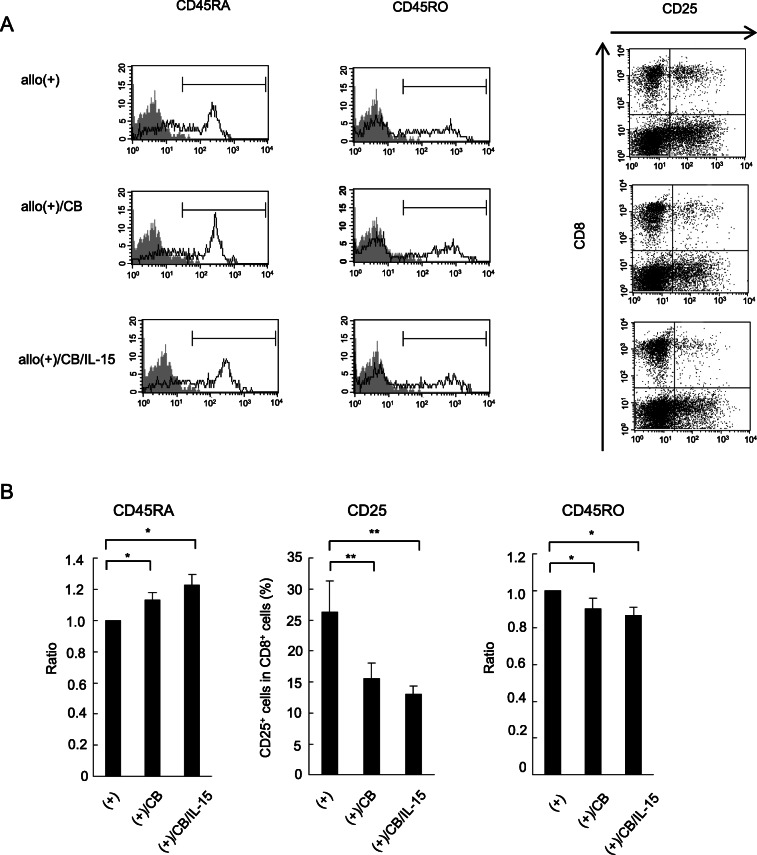
The percentage of CD45RA^-^, CD45RO^-^, and CD25-positive cells in CD8^+^ lymphocytes. PBMCs were harvested from the three groups, representing allogenic stimulation, CB exposed with allogenic stimulation, and CB exposed with allogenic stimulation and IL-15, and assayed for the percentage of CD45RA^-^, CD45RO^-^, and CD25-positive cells using FCM. **a** Representative histograms of cell surface CD45RA and CD45RO expressed on CD8^+^ lymphocytes. Representative dot plots of CD25 versus CD8 on PBMCs. A non-stained control (gray) is shown in each panel. **b** Cumulative data showing percentage of CD45RA^-^, CD45RO^-^, and CD25-positive cells in CD8^+^ lymphocytes. Data showing the ratio of each group to the allogenic-stimulated control was calculated and compared among the groups for the percentage of CD45RA^-^ and CD45RO-positive cells in CD8^+^ cells. Data represent the mean + SD from three independent experiments using PBMCs. Significant differences are indicated by asterisks (^*^p < 0.05, ^**^p < 0.01). (+), the culture with allogenic PBMCs without CB; (+)/CB, the culture with allogenic PBMCs and CB; (+)/CB/IL-15, the culture with allogenic PBMCs and both CB and IL-15

In an effort to determine whether asbestos-induced suppression of naïve CD8^+^T cell differentiation into effector/memory cells could be restored in response to exogenously added IL-15, the percentage of cells positive for the following markers was measured at day 7 after the MLR: CD45RA and CD45RO, expressed on naïve and effector/memory cells, respectively [19, 20], as well as CD25 cells, expressed on activated cells [21]. Exposure to CB resulted in an increase in CD45RA^+^ naïve cells in CD8^+^ lymphocytes stimulated with allogenic PBMCs, and a decrease in CD45RO^+^ effector/memory cells and CD25^+^ activated cells in CD8^+^ lymphocytes (Fig. 3a and b). This is consistent with our previous findings in that the present study could also demonstrate suppressed differentiation into CTLs following exposure to asbestos [13]. Despite the complementary effect of IL-15 on CD3^+^CD8^+^ cell number as mentioned above, suppression of increased levels of CD25^+^ and CD45RO^+^ cells in CD8^+^ lymphocytes and suppressed decrease in CD45RA^+^ cell levels were not restored by the addition of IL-15. These results indicate that appropriate differentiation of CTLs following exposure to CB during the MLR was not achieved by exogenously added IL-15.

### Effect of IL-15 on percentage of granzyme B-positive cells in CD8^+^ lymphocytes

**Fig. 4:**
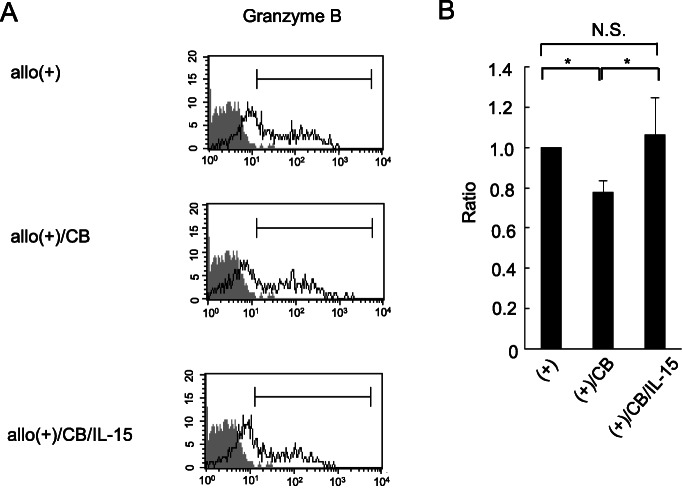
The expression level of granzyme B in CD8^+^ lymphocytes. PBMCs were collected from the three experimental groups: cultured with allogenic stimulation, cultured with allogenic stimulation under CB exposure, and cultured with allogenic stimulation under exposure to CB in media supplemented with IL-15. The expression of granzyme B was analyzed by FCM. **a** Representative histograms showing intracellular granzyme B expression levels in CD8^+^ lymphocytes. Gray-colored histogram shows a non-stained control in each panel. **b** In each experimental group, the percentage of granzyme B-positive cells in CD8^+^ cells was shown as the ratio to the allogenic-stimulated control. Data are obtained from three independent experiments and are shown as the mean + SD. Significant differences are marked with asterisks (^*^p < 0.05). No significant difference is indicated by N.S.. The control group cultured with allogenic PBMCs without CB is represented by (+), the group cultured with allogenic PBMCs under exposure to CB is represented by (+)/CB, and the group cultured with allogenic PBMCs under CB exposure with IL-15 is represented by (+)/CB/IL-15

We examined intracellular granzyme B in CD8^+^ lymphocytes. Granzyme B is a representative mediator of target cell death accomplished by CTLs. Allogenic stimulation induced an increase in the percentage of granzyme B^+^ cells in CD8^+^ lymphocytes, whereas exposure to CB suppressed this increase in the percentage of granzyme B^+^ cells, which is consistent with our previous findings [13]. The addition of IL-15 could diminish that suppression, whereby the percentage of granzyme B^+^ cells did not differ between the control and IL-15-treated culture with CB. These results indicate that exogenously added IL-15 could fully restore the asbestos-induced decrease in percentage of granzyme B^+^ cells (Fig. 4a and b).

### Effect of IL-15 on induction of granzyme B in proliferating and non-proliferating CD8^+^ lymphocytes

**Fig. 5:**
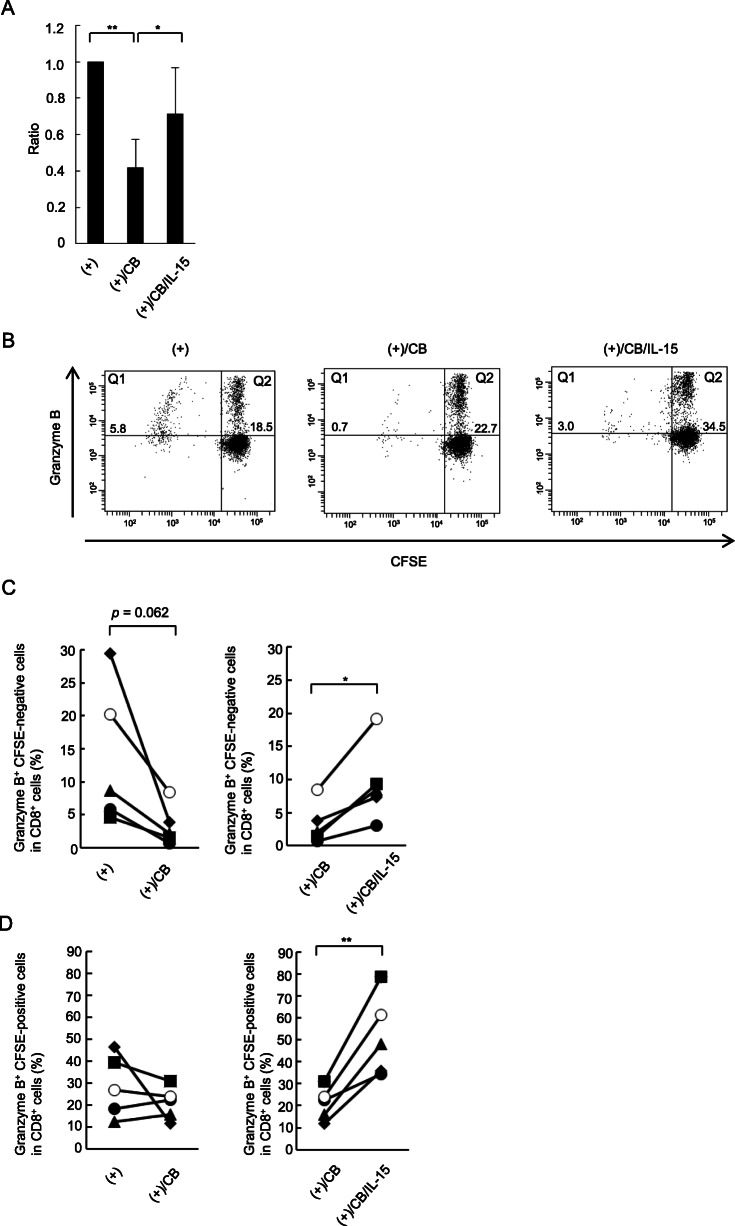
Proliferating and non-proliferating CD8^+^ lymphocytes. PBMCs were collected in each of the two experimental groups: cultured with allogenic stimulation under CB exposure, and cultured with allogenic stimulation under exposure to CB and IL-15. The expression level of granzyme B in proliferating and non-proliferating CD8^+^ lymphocytes, represented by CFSE-negative and CFSE-positive, respectively, were analyzed by FCM. **a** In each experimental group, the percentage of CFSE-negative cells in CD8^+^ cells is shown as the ratio to the allogenic-stimulated control. Data are obtained from five independent experiments using PBMCs and are shown as the mean + SD. Significant differences are marked with asterisks (^*^p < 0.05, ^**^p < 0.01). **b** The dot plots with the parameters of granzyme B and CFSE were used to define granzyme B^+^ cells in proliferating (Q1) and non-proliferating (Q2) populations. The gated population of the CD8^+^ subset was employed for the plots. **c** The granzyme B expression level was shown in proliferating and non-proliferating CD8^+^ cells. Data are obtained from five independent experiments using PBMCs. The data are shown with the same symbol for each experiment. The control group cultured with allogenic PBMCs without CB is represented by (+), the group cultured with allogenic PBMCs under exposure to CB is represented by (+)/CB, and the group cultured with allogenic PBMCs under CB exposure with IL-15 is represented by (+)/CB/IL-15

As mentioned above, although the asbestos-induced decrease in number of CD8^+^ lymphocytes following allogenic stimulation was restored by the addition of IL-15, the effect was incomplete (Fig. 2). Therefore, we set out to determine whether the restored increase in the number of CD8^+^ lymphocytes induced by the addition of IL-15 might be due to recovery of cell proliferation. Additionally, we examined whether the restored increase in granzyme B^+^ cell levels induced by the addition of IL-15 might be accompanied by the recovery of cell proliferation. PBMCs were stained using CFSE before the MLR to detect CFSE-negative proliferating cells. After 7 days of the MLR, PBMCs were collected and stained using granzyme B and CD8 antibodies (Abs). Exposure to CB suppressed the proliferation of CD8^+^ lymphocytes stimulated with allogenic PBMCs, as was found in our previous studies [13]. As shown in Fig. 5a, addition of IL-15 significantly restored the asbestos-induced suppression of CD8^+^ lymphocyte proliferation during the MLR; however, the restored level was lower than that of the control culture without CB. As shown in Fig. 5 b–d , exposure to CB tended to result in a decrease in the percentage of CFSE-negative (proliferating) and granzyme B-positive cells (*p* = 0.062), although the CFSE-positive (non-proliferating) and granzyme B-positive cells in CD8^+^ lymphocytes did not decrease following exposure to CB. Additionally, the percentage of both proliferating and non-proliferating granzyme B-positive cells upon asbestos exposure increased significantly following the addition of IL-15. These results indicate that exogenously added IL-15 led to partial recovery of the asbestos-induced suppression of cell proliferation, and furthermore restored and enhanced intracellular granzyme B in both proliferating and non-proliferating CD8^+^ lymphocytes exposed to asbestos.

## Discussion

Previously, we reported that asbestos exposure suppressed the differentiation of mature CTLs and was accompanied by a decrease in the proliferation of immature CTLs [13]. Then we examined the effect of IL-2 addition on asbestos-induced suppression of CTL induction [14]. Although exogenously added IL-2 suppressed the decrease in intracellular granzyme B, the effect was only partial. Furthermore, the addition of IL-2 could not restore other alterations in cell numbers of CD3^+^CD8^+^ or expression levels of cell surface markers. In the present study, we found that addition of IL-15 led to full recovery of the percentage of granzyme B and partial recovery of proliferation of CD8^+^ lymphocytes in contrast to IL-2 addition, although it did not restore the percentage of CD45RA^+^, CD45RO^+^, or CD25^+^ cells in CD8^+^ lymphocytes after the MLR. These findings indicate that supplementation with IL-15 has the potential to improve the suppressed function of CTLs following exposure to asbestos, although it did not restore the asbestos-induced suppression of mature CTL differentiation following antigen stimulation. Thus, the present study demonstrated that IL-15 is more effective in the recovery of cell proliferation and granzyme B levels following asbestos-induced suppression of CTL induction compared with IL-2.

Unfortunately, secreted IL-15 was not detected via ELISA in the present study, although it is extremely difficult to detect secreted IL-15 protein in the supernatants of cultured cells that express IL-15 mRNA in the published study [22]. Therefore, we could not conclude that an insufficient level of IL-15 is not the predominant cause for the suppressed induction of CTLs following asbestos exposure. Nevertheless, the present study demonstrated that CTLs induced upon exposure to asbestos possess dysfunctional machinery that can be partly compensated by IL-15 supplementation. It is known that asbestos fibers inhaled occupationally or environmentally accumulate in lymph nodes [23, 24], which means that our observation regarding dysfunction in CTLs caused by asbestos could also be relevant to *in vivo* conditions. Several studies using animals have demonstrated the relationship between IL-15 deficiency and malignant diseases. HER2/neu transgenic mice with homozygous knockout of IL-15 (IL15KO/NeuT mice) were compared with IL-15 wild-type HER2/neu transgenic mice (NeuT) with respect to mammary carcinogenesis [25]. IL15KO/NeuT mice showed significantly earlier mammary cancer onset compared with NeuT mice. CD8^+^ lymphocytes were significantly lower in IL15KO/NeuT mice compared with mice with wild-type IL-15, which shows similarity in the suppressed response of CD8^+^ lymphocytes upon exposure to CB asbestos. As shown in another study [26], IL-15^-/-^ mice have increased lung metastasis, whereas IL-15 TG mice and IL-15-treated C57BL/6 mice have decreased lung metastasis compared with control. On the basis of those findings, it is possible that IL-15-deficient mice might show an increased incidence of mesothelioma following exposure to asbestos. Future investigations concerning IL-15 gene expression and signaling upon exposure to asbestos and the effect of IL-15 deficiency on mesothelioma using *in vitro* and *in vivo* experiments should provide critical information of the relationship between IL-15 and mesothelioma caused by asbestos.

Although the percentage of CD3^+^CD4^+^ and CD3-negative cells as well as CD3^+^CD8^+^ cells did not decrease upon exposure to CB (Fig. 2a, c, and e), exposure to CB caused a significant decrease in the number of CD3^+^CD4^+^ and CD3-negative cells as well as CD3^+^CD8^+^ cells (Fig. 2d and f), indicating that asbestos exposure caused damage in CD3^+^CD8^+^ cells and other cells. In contrast, the effect of IL-15 addition differed among the cell populations examined. The addition of IL-15 significantly and partially restored the asbestos-induced decrease in the number of CD3^+^CD8^+^ cells, but not of CD3^+^CD4^+^ or CD3-negative cells (Fig. 2b, d, and f). These results suggest that restoration of the asbestos-induced decrease in the number of CD8^+^ T cells might depend upon direct action of IL-15 on those cells rather than any indirect action mediated by CD3^+^CD4^+^ or CD3-negative cells. Those findings are consistent with a previous study which showed that CD8^+^ T lymphocyte levels consistently decreased relative to CD4^+^ T lymphocytes in both spleen and lymph nodes of *IL-15Rα*^*−/−*^ mice [27]. IL-15 is regarded as a key cytokine not only of CD8 T cells, but also of DC, macrophage, NK, and NKT cells [28, 29]. A discussion of this issue is described later.

As described above, the altered expression of cell surface markers was not restored by exogenously added IL-15. On the other hand, treatment with IL-15 led to recovery of CD8^+^ T cell proliferation and the asbestos-induced decreasing trend in percentage of granzyme B^+^ cells in proliferating CFSE-negative CD8^+^ cells, which were partially and completely restored, respectively. This led us to postulate that asbestos-induced suppression of CTL function might be restored by supplementation with IL-15, which is not the same as simply providing conditions without asbestos. In fact, in addition to contributing towards the generation of primary and memory antigen-specific CD8^+^ T cells [15], IL-15 is known to induce antigen-independent expansion in both naïve as well as non-naïve CD8^+^ T cells, where naïve cells showed down-modulation of CD45RA, while those non-naïve showed up- or down-modulation of CD45RA [30]. Moreover, IL-15 mediates antigen-independent cell proliferation to maintain memory-phenotype CD8^+^ T cells [31]. Thus, IL-15 contributes to augmentation of CTL functions beyond the framework of naïve and memory cell populations. These findings have contributed towards efforts aimed at the understanding of the influence of exogenously added IL-15 on the MLR following exposure to asbestos. In preliminary experiments, we confirmed that allogenic stimulation increased levels of proliferating CFSE-negative granzyme B^+^ cells rather than non-proliferating cells. Additionally, exogenously added IL-15 almost doubled the percentage of non-proliferating CFSE-positive granzyme B^+^ cells in asbestos-exposed cultures with allogenic stimulation compared with control cultures subjected only to allogenic stimulation in 4 of the 5 experiments. Thus, allogenic stimulation induced proliferating granzyme B^+^ cells, whereas IL-15 supplementation induced non-proliferating granzyme B^+^ cells in the culture with allogenic stimulation following exposure to asbestos. Therefore, it is possible that IL-15 might induce a different response from antigen-mediated proliferation given the increase in granzyme B during the MLR.

CD8^+^ lymphocytes from cultures treated with IL-15 and asbestos showed the same level of granzyme B as those cells from cultures subjected only to allogenic stimulation, as shown in Fig. 4. Treatment with IL-15 also led to partial increases in proliferation of CD8^+^ lymphocytes. Tamang and coworkers reported that CD8^+^ T cells can be activated to express granzyme B and engage in proliferation by treatment with IL-15 in the absence of antigens. Such antigen-independent activation of T cells is known as “bystander-activation”, and the mechanism involved in this process might have achieved recovery of granzyme B expression and partial recovery of CD8^+^ lymphocyte proliferation in cultures supplemented with IL-15 [32]. Additionally, as shown in Fig. 5, most of the CD8^+^ lymphocytes in the culture comprised non-proliferating cells, which exhibited an increase in the percentage of granzyme B^+^ cells in response to treatment with IL-15. This finding suggests possible activation of lymphocytes mediated through IL-15 stimulation of other cell populations including monocyte/macrophage, dendritic, NK, and NKT cells. As mentioned above, IL-15 can function in a variety of cell types and cell populations including CD8^+^ T cells. In fact, IL-15 plays a critical role in the development and survival of NK and NKT cells [29], and loss of IL-15 or IL-15Rα results in deficiency of NK and NKT cells [33]. Additionally, *in vitro* experiments have shown that addition of IL-15 restored IL12Rβ1 gene expression in dendritic cells derived from IL-15KO mice [34]. Those macrophage, dendritic, NK, and NKT cells activated by exogenous IL-15 might contribute to enhanced function in non-proliferating CD8^+^ lymphocytes as well as proliferating cells in an indirect manner. Further investigation of cell populations other than CD8^+^ lymphocytes and of the functional properties of those populations will contribute towards an understanding of the mechanism of the suppressive effect of asbestos exposure on the differentiation of CTLs.

IL-7 also plays a role in various CTL functions as well as IL-2 and IL-15. All of these are members of the common gamma chain (γc) receptor family and share the same signaling machinery with JAK1/JAK3. However, those cytokines act differently at each stage of the immune response [16]. IL-2 and IL-15 function at the shared stage of clonal expansion of T cells, whereas IL-7 plays a role in homeostatic expansion of naïve T cells. Additionally, both IL-15 and IL-7 contribute to proliferation and survival of memory T cells, and IL-15 supports DC-T cell interactions in initiation of the immune response. In our previous study, it was found that exogenous supplementation of IL-2 could augment the cytotoxicity of asbestos-exposed CD8^+^ lymphocytes and percentage of granzyme B^+^ cells during MLR culture, which represents an *in vitro* model for the immune response that includes initiation and clonal expansion, but not expansion of naïve cells or maintenance of memory cells. Additionally, CD122 and γc are shared between IL-2 and IL-15. All of the aforementioned data suggest that IL-15 is an appropriate target of investigation in studies together with IL-2 supplementation. Therefore, the present study involved the use of exogenous supplementation of IL-15 during MLR cultures exposed to asbestos.

## Conclusion

Our present investigation demonstrated that addition of IL-15 completely restored the expression of granzyme B and partially restored the proliferation of asbestos-exposed CD8^+^ lymphocytes. This effect was greater than that observed with the addition of IL-2, suggesting that CTLs induced upon exposure to asbestos possess dysfunctional machinery that can be partly compensated by IL-15 supplementation. The finding has motivated us to engage in efforts to identify molecules that could be employed to improve suppressed CTL immune responses resulting from exposure to asbestos. This approach could facilitate delineation of the mechanism responsible for asbestos-induced suppression of CTL function. These issues will be examined in future studies.

## Data Availability

The data used to support the findings of this study are available from the corresponding author upon reasonable request.

## Abbreviations

MLR: Mixed lymphocyte reaction
CTLs: Cytotoxic T lymphocytes
IL-2: Interleukin-2
IL-15: Interleukin-15
PBMCs: Peripheral blood mononuclear cells
CFSE: Carboxyfluorescein diacetate succinimidyl ester
DCs: Dendritic cells
CB: Chrysotile B
Abs: Antibodies
PC5: Phycoerythrin-cychrome 5
FITC: Fluorescein isothiocyanate
PE: Phycoerythrin
FCM: Flow cytometry
RPE: R-phycoerythrin
